# Dr. Bernard Ramsay Craig Christie

**Published:** 1911-04-29

**Authors:** 


The death is announced of Dr. Bernard Ramsay Craig Christie, suddenly, at the age of forty-one. He studied at Edinburgh, Glasgow, and Dublin, having gained the M.B., C.M. in 1897, and graduated M.D. of Edinburgh in 1901. His appointments included that of resident house physician and surgeon at the Glasgow Royal Infirmary, of clinical clerk at the Royal Hospital for Sick Children, Edinburgh, and formerly of assistant medical officer at Gartloch Asylum and Hospital.

